# Latent profiles of emotion regulation among university students: links to repetitive negative thinking, internet addiction, and subjective wellbeing

**DOI:** 10.3389/fpsyg.2024.1272643

**Published:** 2024-04-10

**Authors:** Joana Oliveira, Susana Pedras, Richard A. Inman, Sofia Marques Ramalho

**Affiliations:** Centro de Investigação em Psicologia para o Desenvolvimento (CIPD) [The Psychology for Development Research Center], Instituto de Psicologia e Ciências da Educação (IPCE), Universidade Lusíada, Porto, Portugal

**Keywords:** emotion regulation, latent profile analysis, internet, intrusive thoughts, wellbeing, university students, person-centered

## Abstract

Recent years have seen a deterioration in the mental health of university students and notable surge in the need for psychological support. Due to its links to psychopathology and high-risk behaviors, difficulty in emotion regulation frequently serves as a transdiagnostic dimension. This cross-sectional study used a person-centered analytical approach (latent profile analysis; LPA) to identify groups of Portuguese university students with similar profiles of emotion regulation difficulties (*N* = 261; *M*_age_ = 22.5 ± 1.2 years; *n* = 213 female) and describe how these groups differ in their presentation of repetitive negative thinking, internet addiction, and subjective wellbeing. The analyses identified four latent profiles: 14.5% of students showed global dysregulation (the Low Emotion Regulation Profile), 23% were moderately dysregulated with elevated problems in goal-directed behavior (the Moderate Emotion Regulation Profile), 8% showed specific difficulties with low emotional awareness and clarity (the Low Insight Profile), and 54.4% showed adaptive emotion regulation (the High Emotion Regulation Profile). As anticipated, the Low Emotion Regulation Profile had the lowest subjective wellbeing and the highest prevalence of repetitive negative thinking and internet addiction. Students with a Low Insight Profile also showed low subjective wellbeing, but less repetitive negative thinking compared to the Low Emotion Regulation Profile. Our findings suggest that interventions aimed at improving health and wellbeing among university students should consider each student’s unique set of emotion regulation difficulties, rather than focusing on particular strategies. Further research may help determine whether emotion regulation profiles can serve as predictive indicators of varying mental health trajectories and subjective wellbeing in university students.

## 1 Introduction

In recent years, researchers have become increasingly interested in the role of emotion regulation in human functioning. Numerous studies on this topic have shown, for example, that difficulties in emotion regulation underpin various psychological disorders and other negative psychological outcomes (Gross and John, 2003; Gross and Jazaieri, 2014; Cludius et al., 2020), leading many researchers to now consider emotion dysregulation a major transdiagnostic risk and maintenance factor (Aldao et al., 2010; Aldao and Nolen-Hoeksema, 2010). Conversely, the ability to regulate emotions effectively has been linked to mental health and wellbeing (Richard-Sephton et al., 2023). Given the importance of emotion regulation for understanding adaptive and maladaptive human functioning, this study aims to characterize differences in emotion regulation within a sample of university students—a population known to be at a high risk for mental health difficulties (Storrie et al., 2010; Royal College of Psychiatrists, 2011; Sivertsen et al., 2019)—and to understand how any such differences relate to indicators of healthy and unhealthy functioning.

### 1.1 Emotion regulation: the need for person-centered approaches

Emotion regulation is a complex and multifaceted phenomenon comprised of related-yet-separable strategies and processes (Gratz and Roemer, 2004). According to Gross’s Process Model, emotion regulation involves modifying (in terms of intensity, duration, and/or quality) or maintaining emotions to suit individual goals, and therefore has the primary function of adjusting emotional reactions to be adaptive (Gross, 2015). To achieve one’s goals through emotion regulation, individuals can employ myriad strategies categorized as situation selection, situation modification, attentional deployment, cognitive change, and response modulation strategies. Such strategies can be used consciously and intentionally, but also automatically and unconsciously. Effective use of ‘adaptive’ emotion regulation strategies, such as recognizing and appropriately expressing emotions, has been associated with enhanced physical and mental health outcomes and improved academic performance (Gross and Thompson, 2007; McLaughlin et al., 2011). Conversely, the use of ‘maladaptive’ emotion regulation strategies, such as rumination, has been linked to increased stress, anxiety, and depression, with adverse effects on wellbeing (Schraub et al., 2013; Valenzuela et al., 2020; Chen et al., 2022). However, an important consideration is that strategies are not universally maladaptive or adaptive, with all types of strategies potentially being adaptive or maladaptive in certain contexts (Kashdan et al., 2012).

Given these findings, studies focused on single strategies are unlikely to capture the complex processes through which emotion regulation more broadly influences functioning and psychosocial outcomes. Consequently, it is necessary to adopt person-centered approaches to analyze emotion regulation as an acknowledgment that its various dimensions can be configured differently across people (e.g., McGlinchey et al., 2021). Traditional variable-centered analytic approaches, using methods like regression or correlation, focus on describing how variables are associated *on average* assuming that the dynamics among variables apply in the same way for everybody (Magnusson, 2003). In contrast, person-centered analytic approaches focus on the system of interacting variables, assuming that the dynamics among a set of indicators are heterogeneous within a population (Laursen and Hoff, 2006). One such person-centered approach is Latent Profile Analysis (LPA; Morin et al., 2016). LPA aims to identify relatively homogenous subgroups (referred to as latent profiles) within a sample, each associated with a distinct configuration of indicators. As a model-based procedure, LPA is more flexible than other person-centered methods such as cluster analysis, and has the advantage of providing fit indices that allow for informed decisions about the number of underlying subgroups (Marsh et al., 2009).

Various studies in the last 10 years have applied person-centered approaches, including LPA, to investigate emotion regulation. Some have had the objective to identify emotion regulation profiles in clinical populations and formed profiles using the six validated domains of the Difficulties in Emotion Regulation Scale (DERS; Gratz and Roemer, 2004): difficulties in accepting emotional responses, difficulties in engaging in goal-directed behavior, difficulties controlling impulses, lack of emotional regulation strategies, lack of emotional awareness, and lack of emotional clarity. Typically, these studies have identified only a small number of profiles, potentially due to their relatively small sample sizes. For example, a study of 315 patients with eating disorders identified just two profiles that differentiated between individuals with global dysregulation and low impairment (Riddle et al., 2023). However, other studies have found additional profiles, such as a study of 156 inpatients with borderline personality disorder, which found an “emotionally aware” profile along with low impairment and global dysregulation profiles. Patients in this group had specific difficulties with goal-directed behavior, impulse control, and access to strategies, but not in emotional awareness and clarity (Rufino et al., 2017).

There are fewer studies on this topic with non-clinical samples, although interest in the prevalence of emotion dysregulation in the general population is growing (Marwaha et al., 2013), and they have often had similar results. A large study of 1,165 young adults (average late 20s) by Pinto et al. (2022), for instance, also identified three profiles representing (a) low impairment, (b) global dysregulation, and (c) difficulties with goal-directed behavior, impulse control, and access to strategies in the presence of high emotional awareness (an “emotionally aware” profile). More recently, LPA was used to extract latent emotion regulation profiles in university students, although we note the sizes of the two independent samples used in this study were small for this type of analysis (*n*s < 200; Chesney et al., 2019). Profiles representing low, intermediate, and high emotion regulation ability have also been identified in adolescents (McGlinchey et al., 2021).

### 1.2 Differences among emotion regulation profiles

Beyond identifying subgroups of individuals with distinct emotion regulation profiles, it is useful, and indeed typical in person-centered studies, to compare emergent groups to understand their connection with healthy and/or unhealthy functioning. In the case of emotion regulation in university students, this is because different configurations represent unique ways of adapting and coping with the challenges and difficulties inherent to being a university student. For the present study, we considered two different variables that represent outcomes of emotion regulation ability: subjective wellbeing and internet addiction. Given the need to understand the mechanisms underpinning different emotion regulation profiles (D’Agostino et al., 2017), we also considered a transdiagnostic cognitive process that can hinder emotion regulation: repetitive negative thinking. We summarize each, and their relevance for university students, below:

#### 1.2.1 Subjective wellbeing

Subjective wellbeing, a conceptualization of hedonic experience, can be defined as when an individual feels and evaluates their lives as pleasant and gratifying (Diener et al., 1999). Subjective wellbeing is said to be high when emotional/affective experiences are more positive than negative (‘happiness’) and when, concurrently, an individual’s cognitive judgments about their life circumstances relative to personal criteria like goals and values are good (‘satisfaction’) (Diener et al., 2002). Thus, subjective wellbeing has been widely conceptualized over the last 30 years as having a tripartite structure including positive affect, lack of negative affect, and satisfaction with life (Busseri and Sadava, 2011). Numerous studies have shown that these three components are highly correlated (Keyes et al., 2002) and tend to load on a general subjective wellbeing factor (Jovanovic, 2015). As such, it is common in the literature for studies to calculate and use composite subjective wellbeing scores (e.g., Cloninger and Zohar, 2011).

Promoting health and subjective wellbeing in university students is now widely recognized as being essential for promoting various other outcomes. For example, research suggests that wellbeing is an important antecedent to better academic performance (El Ansari and Stock, 2010), reducing mental health risks (Campbell et al., 2022), fostering personal development, growth, and self-discovery (Yu et al., 2018), and strengthening social connections and support (Cobo-Rendón et al., 2020). Furthermore, promoting subjective wellbeing in university students extends beyond the individual, contributing to a positive and thriving campus culture (Travia et al., 2020). Therefore, subjective wellbeing is pivotal for helping university students excel academically, emotionally, and socially, thus preparing them for success in their university years and beyond.

Given emotion regulation is frequently done to promote a positive rather than negative emotional/affective experience, it is unsurprising that past research has revealed that certain individual ‘adaptive’ and ‘maladaptive’ emotion regulation strategies are differently related to indicators of wellbeing. A classic study by Gross and John (2003), for example, found that cognitive reappraisal was significantly positively correlated with life satisfaction and various indicators of psychological wellbeing. Expression suppression, conversely, was significantly negatively correlated with the same outcomes. However, as far as we are aware, despite research testing the implication of different emotion regulation profiles on clinical variables such as depression and anxiety (Chesney et al., 2019), we are unaware of any study that has examined how such profiles relate to subjective wellbeing.

#### 1.2.2 Internet addiction

Internet usage has seen a remarkable global surge, with one recent estimate classifying 62.5% of all people as internet users (We are Social and Hootsuite, 2022). This widespread use of the internet offers benefits for societies and individuals. For example, internet use can alleviate feelings of loneliness (Phu and Gow, 2019) and has been linked to elevations in university student satisfaction with campus life (Kim et al., 2020). Unfortunately, with the dramatic growth of internet use in the last decades there has also been a significant rise in the proportion of individuals with difficulties regulating their internet use (Pan et al., 2020; Lozano-Blasco et al., 2022). In extreme cases, such difficulties can lead to highly excessive use with characteristics of clinical addiction (Block, 2008), leading some countries to consider it a serious public health issue. Indeed, the concept of internet addition has been widely studied and shown to be comorbid with anxiety and depression (Lee et al., 2008) and other issues such as alcohol dependence (Ko et al., 2008). However, there remains a controversy over whether internet addiction is a true addiction or distinct from other compulsive-impulsive disorders (Yellowlees and Marks, 2007). As such, some researchers use the term “Problematic Internet Use” interchangeably with “Internet Addiction” (Shapira et al., 2003; Montag et al., 2010).

Research indicates that university students may be more susceptible to internet addiction than the general population (Anderson et al., 2017). This trend is concerning because internet addiction is associated with adverse psychosocial outcomes for students, including academic difficulties (Marahatta et al., 2015) and a heightened risk for mental health issues and psychopathology [see meta-analysis of 223 studies by Mannino et al. (2021) and Cai et al. (2023)]. Further empirical evidence underscores the negative impacts of internet addiction on young adults’ subjective wellbeing (Zhao, 2021), with some studies revealing a reciprocal relationship between internet addiction and indicators or illbeing and psychopathology (Zhu et al., 2021).

Several studies have identified a significant positive association between difficulties in emotion regulation and internet addiction among young people [see, for example, the review of 23 studies published between 2010 and 2020 by Gioia et al. (2021)], with internet addiction suggested as a coping mechanism for these difficulties (Caplan, 2010). In other words, emotion dysregulation may be a risk factor for internet addiction because internet use can offer a way of managing or escaping negative emotions. Consistent with this, research on young adults has shown that individuals with internet addiction tend to report greater difficulties in emotion regulation (Evren et al., 2018; Pettorruso et al., 2020). Furthermore, individuals with internet addiction are found to often employ ‘maladaptive’ emotion regulation strategies (Caplan, 2002). Interestingly, some research has suggested that internet addiction could be both a consequence of, and contributor to, emotion dysregulation (Hormes et al., 2014). Despite these insights, there remains a notable gap in the literature: no study has explicitly examined how different emotion regulation profiles in university students relate to their levels of internet addiction.

#### 1.2.3 Repetitive negative thinking

Characterized by its recurrent nature, intrusiveness, and difficulty to disengage from (Ehring and Watkins, 2008), repetitive negative thinking often stems from distressing experiences and can significantly disrupt daily life (Magson et al., 2019). The two most intensively studied expressions of repetitive negative thinking are worry (linked to anxiety) and rumination (linked to depression). Worry has been defined as a predominantly verbal thought activity, which is negatively affect-laden, relatively uncontrollable, and focused on uncertain events with the potential for a future negative outcome (Borkovec et al., 1983). In turn, rumination can be defined as a maladaptive and repetitive thinking style that is activated in response to negative internal and/or external triggers (Nolen-Hoeksema et al., 2008).

Repetitive negative thinking is involved in the maintenance of multiple types of emotional problems and has therefore been described by various researchers as a transdiagnostic cognitive processing style (Ehring and Watkins, 2008; McEvoy et al., 2013). Consistent with this perspective, repetitive negative thinking has been considered as one of the possible mechanisms through which emotion dysregulation occurs and is maintained (Gaynor and Gordon, 2019), and through which individuals may be at increased risk for heightened anxiety, reduced self-esteem, and emotional distress, thereby affecting life satisfaction and wellbeing (Berry et al., 2010; Peixoto and Cunha, 2022). Indeed, rumination, and therefore repetitive negative thinking, is frequently considered in research on emotion regulation as a maladaptive strategy (Kashdan et al., 2012). However, to the best of our knowledge, there are no studies on how emotion regulation profiles differ in repetitive negative thinking.

### 1.3 The present study

University life can be stressful and challenging, leading many students to be at risk of poor mental health (Storrie et al., 2010; Royal College of Psychiatrists, 2011; Sivertsen et al., 2019). Students who can effectively regulate and manage their emotions are expected to be more likely to demonstrate resilience in the face of stress (Troy and Mauss, 2011). However, when emotional responses are poorly regulated students may have difficulty coping, resort to maladaptive behaviors as a means of escape or temporary relief, and experience psychological distress. For these reasons, studying emotion regulation in university students is extremely pertinent for addressing public health and policy concerns about the poor mental health of students.

To this end, it is crucial to recognize that the combinations of strategies used by students to modify and maintain their emotions can differ. Theoretically, university students should not only vary in terms of their overall emotion regulation ability, but also in terms of the configuration of different types of strategies. While different emotion regulation profiles have been identified in diverse clinical and normative samples (McGlinchey et al., 2021; Riddle et al., 2023), there has yet to be a specific investigation of the profiles present in university students. Therefore, the first objective of this study was to apply a person-centered analytic approach (specifically LPA) to a sample of university students. While we refrained from making specific hypotheses about the number and nature of emergent profiles, preferring an exploratory approach, we did anticipate that we would identify a relatively small number of profiles. We then aimed to test how any emergent profiles – reflecting distinct configurations of emotion regulation strategies – differed in terms of subjective wellbeing, internet addiction, and recurrent negative thinking. Again, we did not formulate specific hypotheses, instead opting to describe and understand how different profiles relate to these relevant variables.

## 2 Methods

### 2.1 Participant recruitment and procedures

Data for this study come from a community-based cross-sectional study into internet use and wellbeing in Portuguese university students. Participants were recruited online, with the study advertised on social networks and through academic/personal mailing lists across Portuguese universities. To be eligible for inclusion in the study, participants had to be enrolled in a degree course at a Portuguese university and required an account for at least one social network (WhatsApp, Facebook, Instagram, TikTok, and Snapchat). Proficiency in understanding Portuguese was also required. Students participating in an Erasmus program were not eligible.

Data collection was performed using the online survey software *QuestionPro* in April and May 2023. Before completing the survey, participants were presented with information about the study (including highlighting the right to withdraw) and an online consent form. After ticking a box giving consent, participants were presented with an online survey that took approximately 25 min to complete. Participants were not compensated in any way for their involvement in the study. Ethical approval was obtained from the Ethics Committee of the authors’ university [UL/CE/CIPD/2313].

#### 2.1.1 Sample characteristics

Participants were 261 university students from Portuguese universities across Portugal, of which 175 were undergraduates and 86 were postgraduates (see Table 1). Approximately half of the participants were Psychology students (*n* = 128), with the remainder enrolled in a variety of courses including architecture (*n* = 7), law (*n* = 15), nursing (*n* = 6), engineering (*n* = 9), marketing (*n* = 9) and history (*n* = 4), among others. The sample comprised 213 women (81.6%) and 44 men (16.9%). The mean age across participants was 22.51 years (*SD* = 5.30). On average, the participants reported spending 4.70 h per day (*SD* = 2.16) on their smartphone or computer for leisure (e.g., social media, gaming).

**Table 1:** Sample demographics.

	*M* (*SD*)
Age	22.51 (5.30)
	*n (%)*
Gender	
Male	44 (16.9%)
Female	213 (81.6%)
Other	4 (1.5%)
Education level	
Undergraduate (bachelor’s degree)	175 (67.0%)
Postgraduate (master’s degree or doctorate)	86 (33.0%)

### 2.2 Measures

Participants completed an online survey comprising multiple questionnaires. All survey items were in Portuguese. To minimize order effects, the order of questionnaires within the survey was randomized across participants.

#### 2.2.1 Sociodemographic questionnaire

Participants responded to items that assessed demographic variables including gender (male, female, and other), age, nationality (Portuguese, other), and level of degree currently enrolled in (undergraduate, master, Ph.D.). Participants also indicated the average number of hours they spend per day on various social networks.

#### 2.2.2 Persistent and intrusive negative thoughts scale (PINTS)

The PINTS is a unidimensional 5-item self-report measure of repetitive negative thinking (Magson et al., 2019). Participants respond to statements that evaluate three core features of repetitive negative thinking: (a) repetition, (b) intrusiveness, and (c) difficulty disengaging from such thoughts (Ehring et al., 2011). Participants rate each statement from 1 (*never*) to 5 (*almost always*). The sum scores for the PINTS thus fall within the range of 5–25, with higher scores indicating a higher presence of repetitive negative thinking. The Portuguese version of the PINTS used in this study has been shown to be a reliable (*α* = 0.88) and valid (positive correlation of *r* = 0.52 with a measure of Generalized Anxiety Order; negative correlation of *r* = −0.54 with a measure of Satisfaction with Life) measure in a sample of 432 Portuguese adults (age range = 18–73 years; Peixoto and Cunha 2022). Internal consistency reliability was also good in the present study sample (*ω* = 0.85).

#### 2.2.3 Internet addiction test (IAT)

The IAT is a 20-item self-report questionnaire. As described in its manual (Young, 2017), this test is designed to measure problematic behaviors associated with compulsive use of technology. For each item, participants rate the extent to which they endorse a particular addictive behavior on a Likert-type scale ranging from 0 (*never*) to 5 (*always*) (example item: How often… Do you find that you stay online longer than you intended?). Higher scores reflect greater evidence of internet addiction, but are not necessarily diagnostic of a psychopathological condition. Authors have debated what total score across IAT items would represent a threshold for a clinical diagnosis of “addiction” (Young, 2011) – although for the present study, we treated IAT scores as a continuous variable. The IAT is arguably the most widely validated measure in this domain, with demonstrated psychometric adequacy in various samples globally. The Portuguese version of the IAT used in this study has been shown to be valid (strong positive correlation of *r* = 0.82 with the Beck Depression Inventory-II) and reliable (*α* = 0.90) in a large sample of Portuguese students (age range = 15–39 years; Pontes et al., 2014). Consistent with these past findings, in our study sample, the internal consistency reliability was found to be good (*ω* = 0.88).

#### 2.2.4 Difficulties in emotion regulation scale short form (DERS-SF)

The DERS-SF (Kaufman et al., 2016) is a self-report measure of difficulties in emotion regulation. The 18 items of this scale load on six subscales: (1) Lack of emotional *awareness*; (2) Lack of emotional *clarity*; (3) *Non-acceptance* of emotional responses; (4) Limited access to emotion regulation *strategies*; (5) Difficulties engaging in *goal*-directed behavior; and (5) *Impulse* control difficulties. Participants indicate how much each item applies to themselves from 1 (*almost never*) to 5 (*almost always*). For this study, we calculated sum scores for each subscale. The Portuguese version of the DERS-SF we used was shown to be reliable (α across subscales = 0.70–0.91) in a sample of 646 Portuguese adults (age range = 18–66 years; Gouveia et al., 2022). Consistent with this finding, the internal reliability values of the six subscales in the present study all surpassed the required threshold of acceptability (*ω* = 0.74–0.89).

#### 2.2.5 Positive and negative affect schedule (PANAS)

The PANAS has 20 items, each presenting an adjective that describes an emotional experience (Watson et al., 1988). These items load on two subscales that capture Positive Affect (example item: “Excited”) and Negative Affect (example item: “Afraid”). Participants rate the extent to which they had experienced each emotion in the past 2 weeks on a scale ranging from 1 (*very slightly or not at all*) to 5 (*extremely*). The Portuguese version of the PANAS is widely used and its structure and psychometric adequacy are well supported (e.g., Galinha et al., 2013). For example, in a sample of 348 Portuguese adults aged 18–50 years, both the positive and negative subscales were found to have high internal reliability (*α* = 0.86 and 0.89, respectively) (Galinha and Pais-Ribeiro, 2005). Consistent with other works, in our sample the internal reliability of the positive and negative affect scales was also excellent (*ω* = 0.91 and 0.88).

As is common in studies using the PANAS, we summarized each participant’s perceived affective wellbeing by forming a composite variable (or *Happiness Index;* Josefsson et al., 2011). We calculated this variable by subtracting the mean average of their scores across negative affect items from the mean average of their scores across positive affect items.

#### 2.2.6 The WHOQOL-BREF quality of life assessment

The WHOQOL-BREF is a short-form assessment of quality of life developed by the World Health Organization WHOQOL Group (WHOQOL Group, 1998). Conceptually, quality of life reflects an individual’s perceptions of their life position concerning the cultural and value systems they are part of, as well as their goals, expectations, standards, and concerns. In this way, quality of life captures the cognitive (non-affective) component of wellbeing.

Within this scale, 24 items group in four domains: physical health, psychological health, social relationships, and physical environment. Two additional items request global ratings for quality of life and satisfaction with health. Participants rate all 26 items from 1 to 5, with higher scores reflecting greater perceived quality of life. The Portuguese version of the WHOQOL-BREF has been shown to be reliable (*α* across all items = 0.92) and valid (significantly lower values in hospital patients vs. healthy controls) in a sample of 604 Portuguese adults (Vaz Serra et al., 2006).

To summarize each participants’ cognitive wellbeing, we formed a composite variable (or *Composite Health Index*; Josefsson et al., 2011) by calculating the mean average of their scores across all WHOQOL-BREF items, excluding item 26: “How often do you have negative feelings such as blue mood, despair, anxiety, depression?” We excluded this item because it contaminates the measure with depressive symptomatology (Aigner et al., 2006) and is conceptually closer to affective wellbeing than cognitive wellbeing. In our study sample, the internal reliability consistency across the considered 25 items was excellent (*ω* = 0.92).

### 2.3 Data analysis

#### 2.3.1 Missing data

The study had almost no missing data because of its online format. However, we identified one missing case for one item of the PANAS. For analysis, we replaced this missing using the series mean.

#### 2.3.2 Composite subjective wellbeing index

Subjective wellbeing is a multidimensional construct consisting traditionally of an affective and cognitive component (Diener et al., 1999). To summarize each participant’s subjective well-being, we computed a final composite score by calculating the mean average of their scores on both the *Happiness Index* (from PANAS) and the *Composite Health Index* (from WHOQOL-BREF). This approach has been widely demonstrated to be a valid way to study subjective wellbeing (Cloninger and Zohar, 2011; Josefsson et al., 2011).

#### 2.3.3 Descriptive statistics and correlations

Participant characteristics were analyzed using descriptive statistics, which included calculating means and standard deviations for continuous variables and frequencies (as percentages) for categorical variables. We calculated Pearson’s correlation coefficients to explore the relationships among study variables.

#### 2.3.4 Latent profile analysis

We used a common person-centered mixture modeling analysis (Oberski, 2016) to identify different latent *types*, or subgroups, of individuals based on their standardized mean scores for the six DERS-SF subscales. Specifically, we performed latent profile analysis (LPA) given the continuous nature of these observed variables.

The first step of the LPA process was to decide on the optimal number of latent profiles to explain the observed data. This was done by estimating the parameters, using maximum likelihood estimation, of a series of models that differed in their number of latent profiles (from a 2-profile through to 10-profile solution). Fit indices obtained from each of the models were then compared to determine the model with best fit. Research recommends making this decision based on multiple statistical indices and theoretical considerations (Spurk et al., 2020). For this study, we inspected and compared the following indices: Akaike information criterion (AIC), Bayesian information criterion (BIC; Schwartz, 1978), and sample-size adjusted BIC (SABIC; Sclove, 1987). For these three indices, the model with the lowest value was deemed to have the best fit (Nyland et al., 2007). We also used the bootstrap likelihood ratio test (BLRT; McLachlan and Peel, 2000) to compare models with increasing numbers of latent profiles. The solution prior to an occurring non-significant value (*p* > 0.05) was judged the best fitting. Considering recommendations for best practice we also inspected profile size (Lubke and Neale, 2006).

After deciding on the optimal number of profiles, we sought to validate the profiles by testing how they differed in terms of theoretically relevant outcomes (i.e., internet addiction, repetitive negative thinking, and subjective wellbeing). To do this, we used the *WRS2* package in R to perform a series of robust ANOVAs (Mair and Wilcox, 2020). These robust procedures were employed to counter potentially problematic variances across classes of unequal sizes. As an indicator of effect size, WRS2 calculates an explanatory measure of effect size ξ (Wilcox and Tian, 2011). Values of 0.10, 0.30, and 0.50 correspond to small, medium, and large effects, respectively. Robust ANOVAs were followed by procedures for robust post-hoc comparisons.

## 3 Results

### 3.1 Descriptive statistics and correlational analysis

Table 2 presents descriptive statistics for the study variables. Notably, the full range of possible sum scores was achieved for each DERS dimension. Moreover, values of skewness and kurtosis were indicative that variables had approximately normal univariate distributions (Skew < |2| and Kurtosis < |7|).

**Table 2:** Descriptive statistics for study variables.

	*M*	*SD*	Min	Max	Skew	Kurtosis
DERS subscales						
Lack of emotional awareness	6.92	2.68	3	15	0.45	−0.33
Lack of emotional clarity	6.82	3.06	3	15	0.89	0.16
Difficulties in goal-directed behavior	8.52	3.38	3	15	0.33	−0.94
Difficulties controlling impulses	5.25	2.76	3	15	1.55	2.01
Difficulties accepting emotional responses	6.38	3.07	3	15	0.90	0.05
Lack of strategies	6.31	2.83	3	15	0.89	−0.06
Internet addiction	40.04	10.10	22	74	0.85	0.70
Repetitive negative thinking	17.77	3.95	5	25	−0.12	−0.33
Subjective wellbeing	2.33	0.85	−0.22	4.12	−0.29	−0.38

Correlations among variables are presented in Table 3. As anticipated, difficulties in emotion regulation were positively correlated with internet addiction and repetitive negative thinking. Also as predicted, difficulties in emotion regulation were negatively correlated with subjective wellbeing.

**Table 3:** Correlations among study variables.

Variable	1	2	3	4	5	6	7	8
1. Lack of emotional awareness	1							
2. Lack of emotional clarity	0.36^***^	1						
3. Difficulties in goal-directed behavior	−0.00	0.30^***^	1					
4. Difficulties controlling impulses	0.10	0.31^***^	0.59^***^	1				
5. Difficulties accepting emotional responses	0.09	0.48^***^	0.54^***^	0.51^***^	1			
6. Lack of strategies	0.17^**^	0.53^***^	0.68^***^	0.64^***^	0.62^***^	1		
7. Internet addiction	0.13^*^	0.40^***^	0.39^***^	0.31^***^	0.39^***^	0.46^***^	1	
8. Repetitive negative thinking	0.01	0.33^***^	0.53^***^	0.43^***^	0.41^***^	0.55^***^	0.40^***^	1
9. Subjective wellbeing	−0.33^***^	−0.41^***^	−0.43^***^	−0.36^***^	−0.41^***^	−0.57^***^	−0.40^***^	−0.47^***^

^*^p <0.05, **p <0.01, ***p <0.001.

### 3.2 Latent profile analysis (LPA)

**Figure 1:**
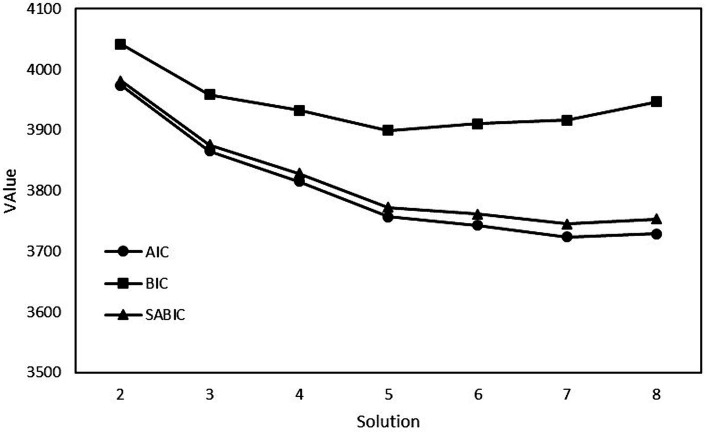
AIC, BIC, and SABIC values plotted as a function of the model.

Table 4 presents fit indices for the series of models estimated. The lowest values obtained for AIC and SABIC were for the 7-profile solution. The lowest value obtained for BIC was for the 5-profile solution. Furthermore, the BLRT *p*-value was non-significant for the 8-profile solution, thus favoring the 7-profile solution. However, the AIC, BIC, and SABIC values indicated substantial flattening after the 5-profile solution (see Figure 1). Entropy decreased after the 5-profile solution, indicating reducing acceptability of classification. However, because the 5-profile solution had two profiles with few participants, we decided that the 4-profile solution was the most parsimonious.

**Table 4:** Fit indices obtained from the latent profile analysis.

	Fit statistics					*N* per profile							
Model	AIC	BIC	SABIC	BLRT_p	Entropy	1	2	3	4	5	6	7	8
2	3974.2	4041.9	3981.6	0.010	0.91	68	193						
3	3865.8	3958.5	3876.1	0.010	0.86	40	74	147					
4	3815.2	3932.8	3828.2	0.010	0.88	38	60	142	21				
5	3756.8	3899.4^*^	3772.6	0.010	0.90	27	62	140	16	16			
6	3743.0	3910.6	3761.6	0.010	0.87	27	42	122	14	22	34		
7	3723.8^*^	3916.3	3745.1^*^	0.010	0.88	26	28	118	15	23	39	12	
8	3729.0	3946.4	3753.0	0.703	0.82	26	32	97	15	21	30	12	28

^*^Smallest value for AIC, BIC, and SABIC.

**Figure 2:**
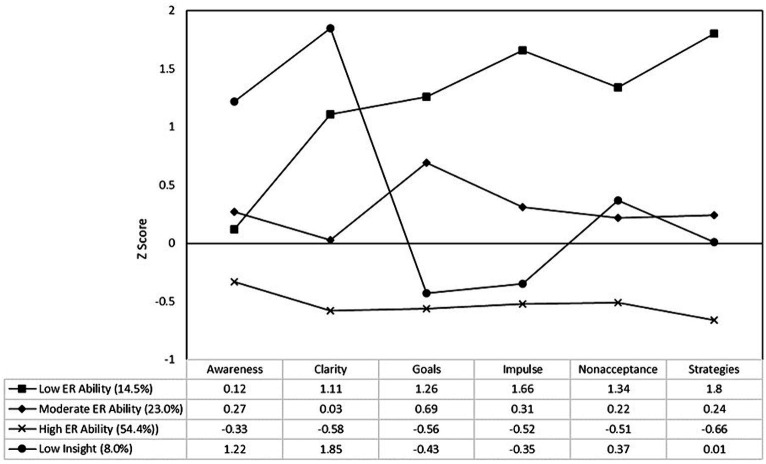
Latent profiles solution for the difficulties in emotion regulation scale.

Three 4-profile solution showed profiles with varying degrees of emotion regulation (ER) ability (Figure 2):

**Low ER Ability Profile (14.5%).** These university students had the most difficulties in goal-directed behavior, impulse control and acceptance of emotional responses, and greatest lack in strategies, with all dimensions at least one standard deviation above the mean (*z* scores >1). This group also showed above average lack of clarity (*z* = 1.11), although they had an approximately average score for lack of awareness (*z* = 0.12).
**Moderate ER Ability Profile (23.0%).** These university students had above average, but less extreme, values for difficulties in goal-directed behavior, difficulties in impulse control, difficulties in acceptance of emotional responses, lack of strategies, and lack of awareness (*z* = 0.22–0.69). They also had an approximately average score for lack of clarity (*z* = 0.03).
**High ER Ability Profile (54.4%).** This largest group of university students had below average values for all six of the DERS domains (*z* = −0.33 – -0.66).
**Low Insight Profile (8.0%).** These university students had a lack of awareness and clarity that was far greater than average (*z* = 1.22 and 1.85 respectively). They also had above average difficulties in acceptance of emotional responses (*z* = 0.37). However, they also have average access to strategies, and scored below average for difficulties in goal-directed behavior (*z* = −0.43) and impulse control (*z* = −0.35).

### 3.3 Comparison of latent profiles

**Figure 3:**
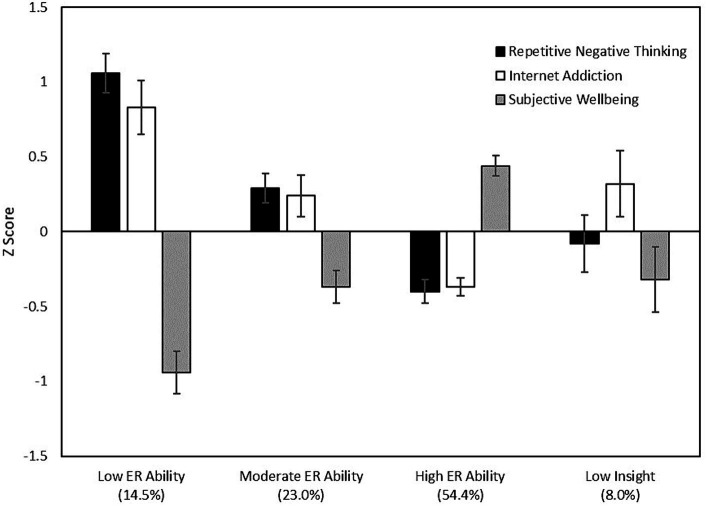
Between-profile differences in repetitive negative thinking, internet addiction, and subjective wellbeing.

Figure 3 presents the mean differences across the profile groups in terms of repetitive negative thinking, internet addiction, and subjective wellbeing. In summary, repetitive negative thinking and internet addiction were most prevalent in the Low ER Ability profile, moderately prevalent in the Moderate ER Ability profile, and least prevalent in the High ER Ability profile. Similarly, subjective wellbeing was lowest in the Low ER Ability profile, at an intermediate level in the Moderate ER Ability profile, and highest in the High ER Ability profile. All these differences were statistically different at *p* < 0.05 (see Table 5 for full ANOVA output). The Low Insight Profile had significantly lower repetitive negative thinking than the Low ER Ability Profile, and significantly lower subjective wellbeing than the High ER Ability Profile.

**Table 5:** Output from the robust ANOVAs.

	*F*	df_1_	df_2_	ξ	95% CI	*Post hoc* comparisons
Repetitive negative thinking	35.84	3	40.9	0.63	[0.50, 0.75]	1 > 2, 3, 4 2 > 3
Internet addiction	16.63	3	37.56	0.47	[0.27, 0.63]	1 > 2, 3 2 > 3
Subjective wellbeing	33.64	3	39.41	0.58	[0.40, 0.71]	1 < 2, 3 2 < 3 3 > 4

1 = Low ER ability profile. 2 = Moderate ER ability profile. 3 = High ER ability profile. 4 = Low insight profile.

## 4 Discussion

For some individuals, the experience of higher education at university can be particularly challenging and adverse to mental health (Storrie et al., 2010; Royal College of Psychiatrists, 2011; Sivertsen et al., 2019). Given the broad role of emotion regulation ability in influencing human adaptive functioning (Aldao et al., 2010), it is a clear construct of interest when looking to understand the factors that explain student experiences at university. However, because emotion regulation is a complex multifaceted phenomenon (Gratz and Roemer, 2004) it is necessary to conduct studies that acknowledge individuals can employ different combinations of strategies that provide different pathways to successful emotion regulation. To this end, the first objective of this article was to use LPA to identify configurations of emotion regulation strategies within a sample of Portuguese university students. From this analysis, we identified four latent profiles that differed quantitatively and qualitatively in terms of the specific difficulties they experienced: (1) the Low ER Ability profile, (2) the Moderate ER Ability profile, (3) the High ER Ability profile, and, (4) the Low Insight profile. The second objective of the article was to test how these profiles differ in terms of repetitive negative thinking, internet addiction, and subjective wellbeing.

An encouraging finding from the study was that the largest profile in terms of membership (including over 50% of the sample) was the High ER Ability profile. Within our sample, these university students had lower-than-average levels of emotion regulation difficulties. As anticipated based on prior studies (e.g., Gross and John, 2003), this profile was associated with the highest levels of reported subjective wellbeing, and the lowest levels of internet addiction and repetitive negative thinking. In other words, we found that the university students most adept at emotion regulation display a set of characteristics indicative of their proficiency in managing and navigating emotions effectively. The DERS profile obtained for this group of students suggests they were distinctly conscious of their emotions and able to recognize and articulate their feelings. This level of self-awareness is instrumental in uncovering the underlying causes of their emotions and helping these students manage stress, adjust to changing circumstances, and recover from adversities they may encounter in higher education. The finding that the High ER ability profile was associated with the highest subject wellbeing and lowest internet addiction score is consistent with various past works emphasizing the role of emotion regulation in student psychological health and academic performance (McLaughlin et al., 2011). The finding that the High ER ability profile was associated with the lowest reported repetitive negative thinking is also consistent with models that present emotion dysregulation as a possible consequence of repetitive negative thinking (D’Agostino et al., 2017; Gaynor and Gordon, 2019).

After the High ER Ability profile, the second largest profile identified was the Moderate ER Ability profile, comprising about a quarter of the sample. These students displayed, for the most part, average difficulties in emotion regulation when compared to others in our sample. However, they were found to have above-average difficulties in goal-directed behavior. This lesser proficiency in emotion regulation was linked to their significantly lower subjective wellbeing, alongside higher rates of internet addiction and repetitive negative thinking, in comparison to the High ER Ability profile. Goal-directed behavior is critical as it encompasses the ability to set specific goals, plan how to achieve them and follow through with the necessary steps to reach those objectives. This ability is closely linked to executive functions, such as planning, organizing, and self-monitoring, which are crucial for academic success and personal development. The distinct challenge with goal-directed behavior observed in the Moderate ER Ability group indicates a particular vulnerability that may translate into lower academic performance, characterized by incomplete assignments, missed deadlines, and stalled progress toward educational objectives. Students struggling specifically with goal-directed behavior may experience difficulties in initiating tasks or a tendency to delay starting assignments and may become frustrated easily when facing obstacles or setbacks, causing them to abandon tasks or goals prematurely. These difficulties could contribute to a cycle of stress, anxiety, and feelings of inadequacy, adversely impacting their overall wellbeing. It is important to note that students with difficulties in goal-directed behavior may not exhibit these behaviors to the same extent, and the severity of the challenges can vary from person to person (Kaufman et al., 2016; Gouveia et al., 2022).

A third profile, which was a notable size, consisted of students categorized under the Low ER profile. These students generally faced more substantial challenges in emotion regulation compared to others in the study sample. This group, characterized by emotional *dysregulation*, notably struggled with understanding their emotions, accepting their emotional states, and accessing effective strategies for emotion regulation. The results also indicated they had difficulties in focusing on goal-directed activities and in controlling their impulses during negative emotional states. However, their level of emotional awareness was approximately average compared to their peers. Consistent with what has been found in the literature about the impact of emotion dysregulation on health, wellbeing and psychological functioning (Schraub et al., 2013; Karaer and Akdemir, 2019; Chen et al., 2022) we found that these students with a Low ER profile had the lowest levels of subjective wellbeing and highest levels of repetitive negative thinking and internet addiction.

Overall, the High ER, Moderate ER, and Low ER profiles broadly differentiate between university students with global dysregulation vs. low or very low impairment. As such, this study is consistent with prior works that have also identified DERS profiles distinguished by their degree of overall level of emotion regulation ability (McGlinchey et al., 2021; Riddle et al., 2023). Additionally, this study introduces a fourth profile, named the Low Insight profile, characterized by extreme difficulties in emotional awareness and clarity. Interestingly, individuals in this group generally scored average for other DERS subscales when compared to others in our sample, and had similar values to the High ER profile in terms of goal-directed behavior and impulse control. Challenges in emotional awareness and clarity involve difficulties in understanding and identifying one’s emotions. These difficulties can hinder a student’s ability to manage their emotions effectively, leading to a sense of discomfort or emotional turmoil without a clear understanding of the emotions causing these feelings. For example, a student may feel overwhelmed and agitated during exams, not realizing the root cause is anxiety about their performance. Emotional clarity is about the precision with which individuals can identify, label and describe their emotions. Students lacking emotional clarity might struggle to articulate their feelings, complicating their ability to navigate emotional situations, such as resolving a disagreement with a friend due to an inability to express their emotions clearly. Despite these challenges in emotional awareness and clarity, the Low Insight profile did not present major indicators of ill-being. This group did not show significant differences in repetitive negative thinking, internet addiction, or subjective wellbeing when compared to the Moderate ER profile. However, it is noteworthy that students in the Low Insight profile reported significantly lower subjective wellbeing compared to those in the High ER profile. This suggests that while specific issues in emotional awareness and clarity may not lead to severe ill-being, they nonetheless contribute to a reduced sense of wellbeing, indicating a nuanced impact on students’ potential for flourishing and thriving at university.

### 4.1 Strengths, limitations, and future directions

The current study contributes valuable insights into the heterogeneity of emotion regulation profiles among university students in Portugal. Nevertheless, it is necessary to acknowledge limitations that influence the generalizability of our findings to the target population. One issue is that the sample was overrepresented by female students, meaning caution is warranted when extrapolating these findings to the broader population of Portuguese university students, which has a more balanced gender distribution. More broadly, given the cultural specificities of Portugal, we advise that identified profiles may differ in samples of university students in different countries. Beyond sample characteristics, we acknowledge that the sample size was relatively small for LPA, meaning that we may not have identified rarer profiles of emotion regulation. Thus, future studies with larger samples are required to replicate this study. Finally, the study used self-report measures that were completed online. Consequently, it may be the case that some participants responded in a way that made them seem favorable, rather than truthfully. Moreover, it may be the case that such individuals were not equally distributed across the emergent profiles.

Future research endeavors should aim to replicate and extend our findings to more diverse populations, encompassing individuals of various age groups and life conditions. Longitudinal studies can provide a deeper understanding of the temporal dynamics and predictive power of emotion regulation profiles considering the assessed variables. Exploring the transdiagnostic effects of interventions targeting emotion regulation strategies on promoting subjective wellbeing holds promise for enhancing personalized strategies for intervention and support in this population. In conclusion, while this study enriches our understanding of emotion regulation among university students, its limitations underscore the need for comprehensive, population-specific studies and prospective research to unveil the nuanced interplay between emotion regulation, mental health, and wellbeing.

### 4.2 Implications for practice

Our study revealed that half of the surveyed students exhibited adaptive and functional emotion regulation, while the other half demonstrated varying degrees of challenges: 14.5% showed global dysregulation, 23% were moderately dysregulated with elevated problems in goal-directed behavior, and 8% showed specific difficulties with low emotional awareness and clarity. This diversity underscores the need for a multifaceted approach to promoting healthy emotion regulation among university students. Indeed, recognizing the diversity in emotion regulation profiles among students necessitates tailored interventions that align with the unique attributes of each profile. To this end, acknowledging that there are no one-size-fits-all solutions, universities may employ various strategies to help develop emotion regulation in their students, and by extension help promote health and wellbeing. Potential solutions for promoting emotion regulation that are supported by empirical evidence include: the provision of counseling services (Wiedermann et al., 2023); the provision of workshops and training sessions focused on emotional intelligence, stress management, and coping skills (Gilar-Corbi et al., 2018); encouraging the practice of mindfulness and meditation, including through smart phone apps (MacDonald, 2021); and the encouragement of journaling as a tool for self-reflection and emotional expression (Hashemi and Mirzaei, 2015). Writing about emotions and experiences can help students gain insight into their feelings (Stapleton et al., 2021), and thus may be especially useful for students with a Low Insight profile.

## 5 Conclusion

This person-centered study reveals that Portuguese university students exhibit a range of emotion regulation abilities. While approximately half of the sample were classified as having strong ER ability, the remainder had configurations indicating moderate ER ability (23%), low ER ability (14.5%), or low emotional insight (8%). Significant variation was observed among these groups of students in terms of repetitive negative thinking, internet addiction, and subjective wellbeing. Notably, students with global dysregulation (low ER ability group) were associated with the worst health and wellbeing outcomes. These findings underscore the need for the development of comprehensive, multi-component programs aimed at enhancing emotion regulation skills in university students. Additionally, our results highlight the importance of adopting person-centered methods to better understand differences in student wellbeing, internet addiction, and repetitive negative thoughts. In sum, this approach helps elucidate the relationship between emotion regulation strategies and their influence on university student wellbeing, offering critical insights for targeted interventions.

## Data availability statement

The raw data supporting the conclusions of this article will be made available by the authors, without undue reservation.

## Ethics statement

This study, involving volunteer human participants, was approved by the Ethics Comission of Universidade LusÍada. The research was conducted in accordance with local legislation and institutional requirements. The participants provided their written informed consent to participate in this study.

## Author contributions

JO: Writing – original draft, Supervision, Methodology, Investigation, Conceptualization. SP: Writing – original draft, Conceptualization. RI: Writing – original draft, Formal analysis, Writing – review & editing, Visualization. SR: Writing – original draft, Supervision, Conceptualization.
